# Volumineuse adénopathie cervicale kystique métastatique d´un microcarcinome papillaire de la thyroïde

**DOI:** 10.11604/pamj.2021.38.400.25732

**Published:** 2021-04-26

**Authors:** Mehdi Hasnaoui, Sana Farhat

**Affiliations:** 1 Department of Otolaryngology-Head and Neck Surgery, Tahar Sfar Hospital, Mahdia, Tunisia

**Keywords:** Lymphadénopathie, kystes, carcinome papillaire de la thyroïde, tomodensitométrie, Lymphadenopathy, cysts, papillary thyroid carcinoma, computed tomography

## Abstract

Cervical cystic lymph node metastasis may be confused with a branchial cyst or a cystic lymphangioma. It may be the only tell-tale sign of a papillary carcinoma of the thyroid gland. We here report the case of a 50-year-old patient presenting with a swelling in the right lower region of the jugular vein and carotid artery which had grown over the preceeding three years. Clinical examination showed renitent swelling. There was no palpable thyroid nodule. Cervical ultrasound showed a fluid cystic lesion measuring 10 cm and containing some septa. It didn’t show any thyroid nodule. Cervical computed tomography (CT) scan showed right laterocervical cystic mass containing peripheral calcifications and pressing the internal jugular vein and the sternocleidomastoid muscle. The patient underwent cervicotomy. Extemporaneal anatomopathological examination showed metastasis from papillary microcarcinoma of the thyroid gland. The patient underwent total thyroidectomy associated with bilateral central compartment and right lateral side compartment dissection. Anatomopathological examination showed nonencapsulated papillary thyroid microcarcinoma measuring 3 mm along its longer axis. The patient had lymph node metastases in the left and right sectors VI and in the right sector IV. The patient underwent two cycles of radioiodine therapy with excellent outcome.

## Image en médecine

Une adénopathie cervicale métastatique kystique peut être confondue avec un kyste branchial ou un lymphangiome kystique. Elle peut être le seul signe révélateur d´un carcinome papillaire de la thyroïde. Nous rapportons le cas d´un patient âgé de 50 ans qui nous a consulté pour une tuméfaction jugulo-carotidienne basse droite évoluant depuis 3 ans. A l´examen, la tuméfaction était rénitente. Il n´y avait pas de nodule thyroïdien palpable. L´échographie cervicale a montré une lésion kystique liquidienne de 10 cm renfermant quelques cloisons. Elle n´a pas révélé des nodules thyroïdiens. La tomodensitométrie cervicale a montré une masse kystique latéro-cervicale droite contenant des calcifications périphériques et refoulant la veine jugulaire interne et le muscle sterno-cléido-mastoïdien. Le patient a eu une cervicotomie. L´examen anatomopathologique extemporané était en faveur d´une métastase d´un carcinome papillaire de la thyroïde. Le patient a eu une thyroïdectomie totale associée à un évidement bilatéral du compartiment central et un évidement du compartiment latéral droit. L´examen anatomopathologique définitif a montré un microcarcinome papillaire de la thyroïde faisant 3 mm de grand axe non encapsulé. Il y avait des métastases ganglionnaires des secteurs VI droit et gauche et du secteur IV droit. Le patient a eu deux cures d´iode radioactif avec une excellente évolution.

**Figure 1:**
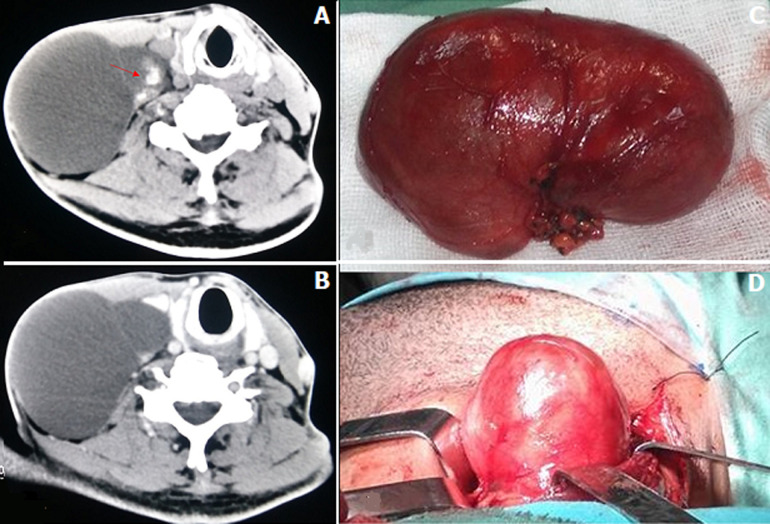
A, B) tomodensitométrie cervicale avec et sans injection du produit de contraste montrant une masse kystique latéro-cervicale droite contenant des calcifications périphériques (flèche) et refoulant la veine jugulaire interne et le muscle sterno-cléido-mastoïdien; C, D) pièce opératoire d´une adénopathie kystique faisant 10 cm de grand axe

